# Repetitive ozone exposure worsens features of muco-inflammatory disease in developed *Scnn1b*-Tg+ mice lungs

**DOI:** 10.3389/ftox.2025.1540468

**Published:** 2025-05-27

**Authors:** Thao Vo, Sonika Patial, Yogesh Saini

**Affiliations:** ^1^ Department of Population Health and Pathobiology, College of Veterinary Medicine, North Carolina State University, Raleigh, United States; ^2^ Division of translational Toxicology, National Institute of Environmental Health Sciences, Research Triangle Park, Durham, United States

**Keywords:** ozone, mice, *Scnn1b*-Tg+, lung, inflammation

## Abstract

**Introduction:**

Ambient exposure to ozone (O_3_), one of the six criteria pollutants, is associated with the exacerbation of respiratory symptoms in individuals with underlying lung diseases. Using *Scnn1b*-Tg+ (Tg+) mice, a widely used model of muco-inflammatory lung disease, we have demonstrated that O_3_ exposure during the early stages of postnatal lung development leads to exacerbated muco-inflammatory outcomes. However, it remains unclear whether O_3_ affects the developed lungs differently than the underdeveloped lungs of Tg+ mice.

**Methods:**

We exposed 3-week-old wild-type (WT) and Tg+ mice to either filtered air (FA) or 0.8 ppm O_3_ for 3 weeks and examined the lung phenotypes 12-16 h post-last exposure.

**Results:**

As compared to FA-exposed WT mice, O_3_-exposed WT mice showed increased bronchoalveolar lavage fluid (BALF) proteins, increased immune cells, increased inflammation, alveolar space enlargement, and tissue consolidation. As compared to FA-exposed WT mice, the FA-exposed Tg+ mice showed increased immune cells, elevated levels of inflammatory mediators, e.g., IL-5, G-CSF, MIP-2, KC, MIP-1α, MIP-1β, IP-10, TNF-α, and IL-17, increased inflammation, alveolar space enlargement, tissue consolidation. As compared to FA-exposed *Scnn1b*-Tg+ mice, O_3_-exposed Tg+ mice had increased total protein, total dsDNA, and phagocytosed lipid contents, in addition to exaggerated granulocytic recruitment, peripheral and bronchiolar inflammation, alveolar space enlargement, and tissue consolidation.

**Discussion:**

Together, our data using Tg+ mice with developed lungs exhibited several findings consistent with previous findings observed in Tg+ neonates. Interestingly, however, as opposed to the previous report in O_3_-exposed neonatal Tg+ mice, where the hallmark features of Tg+ airway disease, i.e., mucus obstruction and expression of major gel-forming mucins (MUC5B and MUC5AC) were found exacerbated by O_3_ exposure, the FA- and O_3_-exposed Tg+ mice with developed lungs exhibited comparable responses. These differential responses suggest that the stage of lung development is an important factor in the modulation of epithelial remodeling following O_3_ exposure.

## 1 Introduction

“Ground level” ozone (O_3_), one of the six criteria gases according to the National Ambient Air Quality Standards (NAAQS), is a global health risk (Cohen et al., 2017). Elevated ground level O_3_ results in increased hospital admissions, which leads to substantial health and economic burdens (Awang et al., 2016; Lin et al., 2008; Malig et al., 2016; Strickland et al., 2010; Tian et al., 2018). O_3_ generation involves the reaction of ultraviolet sunlight with nitrogen oxides (NOx) and volatile organic compounds (VOCs), which can be found in commercial and industrial products (Allen et al., 1997; McDonald et al., 2018; Reinhart, 1993). As a gaseous pollutant, the respiratory tract is the primary target of O_3_ toxicity (Goldstein, 1978; Mehlman and Borek, 1987; Stokinger, 1965). Ambient O_3_ inhalation is linked to respiratory tract inflammation, resulting in coughs, chest tightness and reduced lung function (Bascom et al., 1996). According to the American Lung Association, O_3_ affects children, elderly, and people with pre-existing lung comorbidities. Specifically, O_3_ is known to exacerbate symptoms in patients with ongoing respiratory diseases such as asthma (Khatri et al., 2009), cystic fibrosis (CF) (Farhat et al., 2013; Goss et al., 2004) and chronic obstructive pulmonary disease (COPD) (Strosnider et al., 2019). However, the nature of interactions between O_3_ and diseased lungs is not fully elucidated.

CF is a chronic and progressive hereditary disease that evolves from small airway obstruction with mucus plugging and air trapping, airway inflammation to chronic bacterial infection, bronchiectasis and ultimately death (Grasemann and Ratjen, 2023; Shteinberg et al., 2021). CF transmembrane conductance regulator (CFTR) is a chloride (Cl^−^) channel and regulator of epithelial sodium (Na^+^) ion channel (ENaC), which controls the optimal airway surface liquid layer (ASL) volume and effective mucociliary clearance in the airways (Mall, 2020). In CF, recessive mutations in CFTR gene result in the formation of non-functional CFTR ion channel, which fails to regulate Cl^−^ and bicarbonate (HCO_3_^−^) ions. The Na^+^/Cl^−^ ionic imbalance creates osmotic gradient to draw ASL water into the epithelial cells, which depletes ASL volume and impairs mucociliary clearance (Mall et al., 2010). While CFTR mutated mice do not exhibit features of lung disease (Lewis et al., 2019), the *Scnn1b-*Tg+ mouse overexpresses sodium channel, non-voltage gated 1, beta subunit (*Scnn1b)* transgene in the club cells, which dictates increased ionic concentration of Na^+^ within the epithelial cells and establishes osmotic gradient to draw water from ASL (Mall et al., 2004).

The *Scnn1b-*Tg+ mouse recapitulates the features of human CF lung disease such as mucus hyperconcentration, mucus obstruction, mucous cell metaplasia, susceptibility to bacterial infection and airway inflammation, making it a widely used model for CF (Mall et al., 2008; Zhou et al., 2011). Previously, the *Scnn1b-*Tg+ mouse has been studied in the context of environmental pollutants including cigarette smoke (Engle et al., 2019), secondhand smoke (Lewis et al., 2017), allergens (Fritzsching et al., 2017), and nanoparticles (Geiser et al., 2013; Kim et al., 2022). The effects of O_3_ on muco-inflammatory responses in developing lungs of *Scnn1b-*Tg+ mice were previously reported (Choudhary et al., 2021c). However, it remains unclear whether O_3_ affects the developed lungs differently than the underdeveloped lungs of Tg+ mice. Accordingly, we hypothesized that post-weaning (PND 21 ± 3) repetitive O_3_ exposure will exaggerate inflammatory responses and alter lung pathology in *Scnn1b-*Tg+ mice. Of note, mouse lung development is almost complete at the age of 3 weeks (Bartman et al., 2020; Rackley and Stripp, 2012). Towards this, we repetitively exposed 3-week-old WT and *Scnn1b-*Tg+ (Tg+) weanlings to filtered air (FA) or O_3_ (0.8 ppm) and examine the lung inflammatory responses and pathology after 3 weeks of O_3_ exposure by assessing relevant endpoints, i.e., immune cell recruitment, cytokine analyses, immunohistochemistry, gene expression analyses and pathological features. This study provides interesting insights into the effects of O_3_ on muco-inflammatory responses in Tg+ mice with CF-like lung disease.

## 2 Materials and methods

### 2.1 Mice breeding scheme and animal husbandry

*Scnn1b*-Tg+ mice [*Tg (Scgb1a1-Scnn1b)6608Bouc/J*] on congenic C57BL/6J background were procured from Jackson Laboratory (Bar Harbor, ME). As previously reported, pups were genotyped for *Scnn1b* transgene using polymerase chain reaction (PCR) and were maintained in hot washed, individually ventilated cages on a 12 h dark/light cycle at the Division of Laboratory Animal Medicine (DLAM) at Louisiana State University, Baton Rouge, LA (Lewis et al., 2017). Mice were supplied with food and water *ad libitum* except during filtered air (FA) or ozone (O_3_) exposure (4h/day). All animal procedures were performed under animal protocol approved by the Institutional Animal Care and Use Committee (IACUC) of Louisiana State University.

### 2.2 Ozone and filtered air exposure

*Scnn1b*-Tg+ mice and wild-type (WT) littermates (PND 21 ± 3) were transferred to individual cages with perforated lids without access to food and water and were exposed to either FA or O_3_ [0.822 ± 0.003 (SEM) ppm]. O_3_ exposure was conducted as previously reported (Choudhary et al., 2021c). O_3_ was generated from the O_3_ generator (TSE, Chesterfield, MO). O_3_ concentration, along with chamber pressure, humidity and temperature were displayed as graphs and were monitored throughout the entire exposure period (3 weeks, 5 days/week, 4h/day). Mice are nocturnal and display higher physical activity in nightly conditions (Hawkins and Golledge, 2018). To simulate the real-life scenarios of higher activity phase in humans, mice were transferred to a dark chamber and were exposed to FA or O_3_ during the night cycle.

### 2.3 Tissue harvesting and bronchoalveolar lavage fluid analyses

FA-exposed and O_3_-exposed WT and *Scnn1b*-Tg+ mice were anesthetized via intraperitoneal injection of 2,2,2-tribromoethanol (Millipore Sigma, Burlington, MA). Midline laparotomy was performed to expose and severe inferior vena cava for exsanguination and thoracotomy were performed to expose the lung and trachea. The left main stem bronchus was ligated with suture. The right lung lobes were lavaged with a body weight-adjusted volume of ice-cold Dulbecco’s Phosphate Buffered Saline (DPBS) (Corning, Manassas, VA). Bronchoalveolar lavage fluid (BALF) was centrifuged at 500 × g for 5 min at 4°C. Cell-free BALF supernatant was transferred to a new tube and stored at −80°C for total protein estimation, double-stranded (ds) DNA estimation, and cytokine analyses. Cell pellet was resuspended in 500 µL of fresh DPBS. Total cell counts were determined using a hemocytometer (Bright-Line, Horsham, PA). BALF cytospins were prepared and stained for differential cell counts (Modified Wright Giemsa stain Kit; Newcomer Supply, Middleton, WI). Unlavaged left lung lobe was stored in 10% neutral buffered formalin (NBF) for histopathological and immunohistochemical analyses, and lavaged right lung lobes were snap-frozen and stored at −80°C for gene expression analyses.

### 2.4 Oil-Red-O staining

BALF cytospin slides were prepared and stained for Oil-Red-O staining (Electron Microscopy Science, Hatfield, PA). Cytospin slides were fixed with 10% NBF for 1 h and washed with distilled water twice, then washed with 60% isopropanol for 5 min and dried completely at room temperature (RT). Air-dried cytospin slides were incubated with Oil-Red-O working solution (Oil-Red-O stock diluted with distilled water in 3:2 ratio) for 10 min and washed with distilled water four times. Slides were counterstained with Harris hematoxylin (Millipore Sigma, Burlington, MA) for 1 min, washed with tap water, and then submerged in 0.25% ammonia water for 1 min. Slides were rinsed with tap water and mounted with glycerol jelly medium. Photomicrographs were captured using the ECLIPSE Ci-L microscope with DS-Fi2 camera attachment (Nikon, Melville, NY) for analysis.

### 2.5 Histopathology

Formalin-fixed left lungs were paraffin-embedded and sectioned at 5 μm and processed for histopathological analyses. Hematoxylin and Eosin (H&E) staining (Millipore Sigma, Burlington, MA) was performed to examine the structural and morphological alternations in the lung architecture. Alcian blue/periodic acid-Schiff (AB/PAS) staining (Leica, Buffalo Grove, IL) was performed to access the airway mucus contents and mucous cell metaplasia (MCM) status. All the slides were graded by a board-certified anatomic pathologist in a blinded manner.

### 2.6 Bronchoalveolar lavage fluid analyses

Total protein contents in the cell-free BALF were determined by Bradford assay (Bio-Rad, Hercules, CA). Total dsDNA contents were measured using the Nanodrop 8000 spectrophotometer (Thermo Scientific, Waltham, MA). Cell-free supernatant was assayed with Luminex-XMAP-based assay (MCYTOMAG-70K) according to the manufacturer’s instructions (EMD Millipore, Billerica, MA).

### 2.7 Immunohistochemistry

Immunohistochemical staining for Major Basic Protein (MBP), Lymphocyte antigen 6B.2 (Ly-6B.2), Resistin-like alpha (RETNLA), Matrix Metalloproteinase 12 (MMP12), Mucin 5B (MUC5B), Mucin 5AC (MUC5AC), was performed, as previously reported (Choudhary et al., 2021c), Briefly, formalin-fixed, paraffin-embedded lung sections were deparaffinized with Citrisolv (Decon Laboratories, King of Prussia, PA) and rehydrated to distilled water. Antigen retrieval was performed by incubating sections in Proteinase K (at 37°C for 20 min) for MBP and Ly-6B.2, or by boiling (at 95°C–100°C for 30 min) in citrate buffer for other markers, followed by cooling to RT. 3% hydrogen peroxide was used to quench endogenous peroxidases before blocking step and primary antibody incubation with rat monoclonal MBP primary antibody (MT-14.7.3; Mayo Clinic, Scottsdale, AZ), rat monoclonal Ly-6B.2 primary antibody (MCA771G; Clone 7/4, Bio-Rad, Hercules, CA), rabbit polyclonal MMP12 primary antibody (ab66157; Abcam, Cambridge, MA), rabbit polyclonal MUC5B primary antibody (UNC223; University of North Carolina, Chapel Hill, NC), rabbit polyclonal MUC5AC primary antibody (UNC294; University of North Carolina, Chapel Hill, NC), followed by diluted biotinylated secondary antibodies. The slides were then processed with VECTASTAIN Elite ABC HRP Kit (PK-6101; Vector Laboratories, Burlingame, CA), followed by chromogenic substrate conversion to insoluble colored precipitate using ImmPACT Nova RED HRP substrate Kit (SK-4800; Vector Laboratories, Burlingame, CA). Finally, the slides were counterstained with Gill’s Hematoxylin-I, dehydrated in alcohol solution and mounted with VectaMount mounting media (H-5000; Vector Laboratories, Burlingame, CA). Fiji software (National Institute of Health) was used to determine the positively stained cells (Schindelin et al., 2012).

### 2.8 Gene expression

Gene expression analyses on mRNA harvested from right lungs were performed as previously described (Lewis et al., 2020b).

### 2.9 Statistical analyses

One-way analysis of variance (ANOVA) followed by the Tukey’s *post hoc* test for multiple comparisons was used to determine significant differences among groups. Student’s t-test was used to determine significant differences between FA-exposed and O_3_-exposed *Scnn1b*-Tg+ groups. Outliers were identified and removed using Grubb’s test. Individual data points in scatter plots represent individual animals. All data were expressed as mean ± standard error of the mean (SEM). *P*-value < 0.05 was considered statistically significant. Statistical analyses were performed using GraphPad Prism 8.0.1 (GraphPad Software, La Jolla, CA).

## 3 Results

### 3.1 Ozone exposure increases total protein, double-stranded DNA, and lipid contents in the lung airspaces of *Scnn1b*-Tg+ (Tg+) mice

**FIGURE 1:**
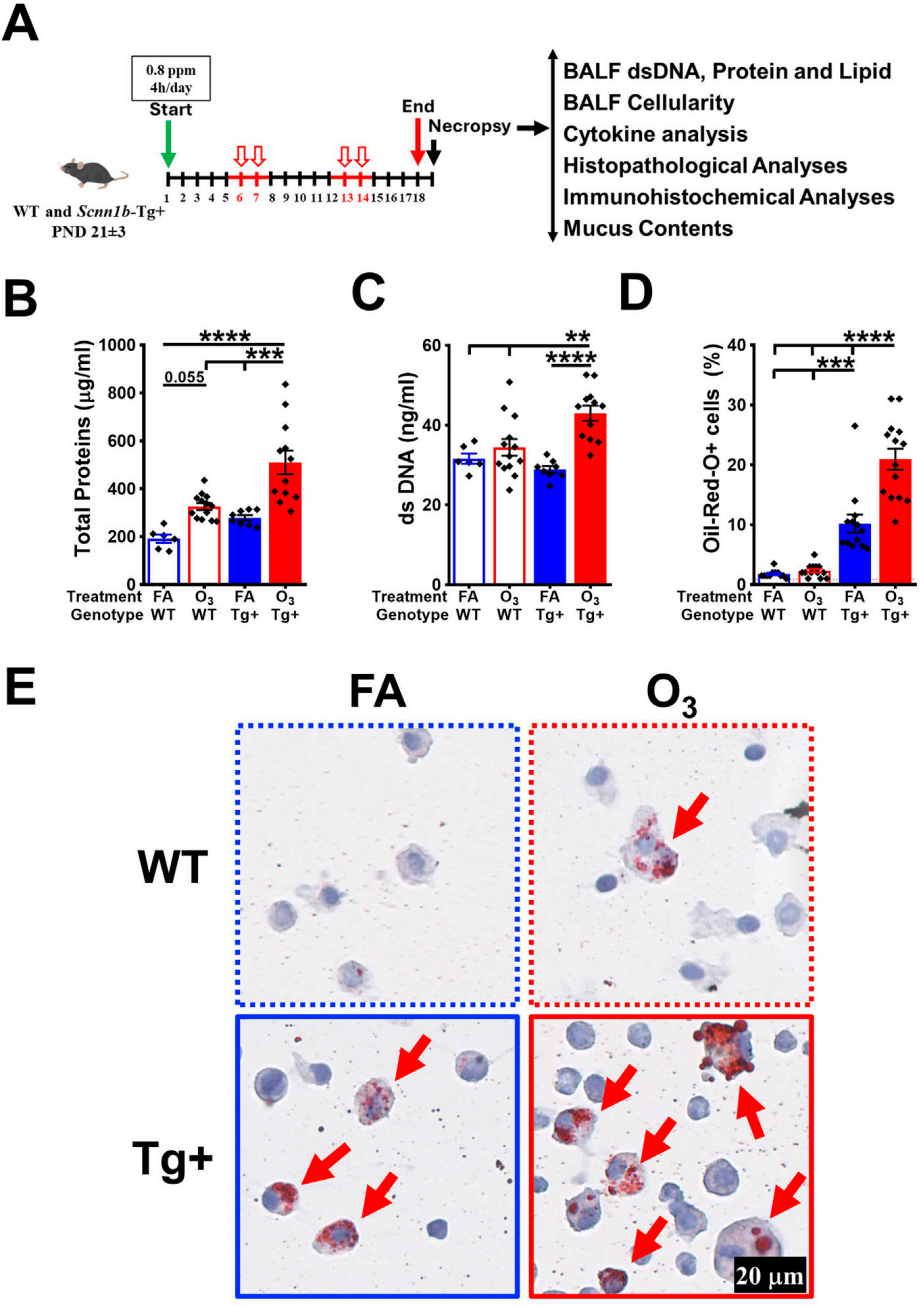
Ozone exposure increases total protein, double-stranded DNA, and lipid contents in the lung airspaces of *Scnn1b*-Tg+ (Tg+) mice*.* **(A)**. Experimental design of ozone (O_3_) exposure in wild-type (WT) and *Scnn1b*-Tg+ mice. **(B)** Total protein concentration (μg/mL) in the bronchoalveolar lavage fluid (BALF) from FA-exposed WT (blue open bar), O_3_-exposed WT (red open bar), FA-exposed *Scnn1b*-Tg+ (blue solid bar), and O_3_-exposed *Scnn1b*-Tg+ (red solid bar) mice. Error bars represent standard error of the mean (SEM), ***p < 0.001, ****p < 0.0001 using one-way ANOVA, followed by Tukey’s multiple comparison *post hoc* test. **(C)** Double-stranded (ds) DNA concentration (ng/μL) in the BALF from FA-exposed WT (blue open bar), O_3_-exposed WT (red open bar), FA-exposed *Scnn1b*-Tg+ (blue solid bar), and O_3_-exposed *Scnn1b*-Tg+ (red solid bar) mice. Error bars represent SEM, **p < 0.01, ****p < 0.0001 using one-way ANOVA, followed by Tukey’s multiple comparison *post hoc* test. **(D)** Percent Oil-Red-O-stained macrophages in the BALF from FA-exposed WT (blue open bar), O_3_-exposed WT (red open bar), FA-exposed *Scnn1b*-Tg+ (blue solid bar), and O_3_-exposed *Scnn1b*-Tg+ (red solid bar) mice. Error bars represent SEM, ***p < 0.001, ****p < 0.0001 using one-way ANOVA, followed by Tukey’s multiple comparison *post hoc* test. **(E)** Representative photomicrographs of Oil-Red-O-stained cytospin made from BALF from FA-exposed WT (blue dotted border), O_3_-exposed WT (red dotted border), FA-exposed *Scnn1b*-Tg+ (blue solid border), and O_3_-exposed *Scnn1b*-Tg+ (red solid border) mice. Red arrows depict lipid-laden macrophages. All photomicrographs were taken at same magnification.

To study the effects of ozone (O_3_) exposure in the lungs of wild-type (WT) and *Scnn1b*-Tg+ (Tg+) mice, Tg+ and littermate WT weanlings (PND 21 ± 3) were exposed to filtered air (FA) or 0.8 ppm O_3_ for 3 weeks (5 days/week, 4h/day) during the night cycle and euthanized within 12–16 h of the final exposure. After necropsy, we assessed bronchoalveolar lavage fluid (BALF) proteins, BALF double-stranded (ds) DNA, BALF lipids, BALF immune cell counts, BALF cytokines, tissue granulocytic infiltration, tissue histopathology, and mucus contents (Figure 1A).

O_3_ exposure has been reported to cause protein leakage into the airspaces, indicative of the disruption of the epithelial-endothelial barrier (Currie et al., 1998; Michaudel et al., 2018; Choudhary et al., 2021c). Moreover, extracellular double-stranded (ds) DNA is an indicator of epithelial injury and inflammation (Kirchner et al., 1996; Joyner et al., 2013). To assess the levels of protein and dsDNA in the lung airspaces of WT and Tg+ mice, we assayed the total protein and dsDNA contents in the cell-free BALF supernatant from FA- and O_3_-exposed WT and Tg+ groups. The total protein contents were increased in the BALF from O_3_-exposed WT versus FA-exposed WT mice, but the differences were not statistically significant (Figure 1B). The total protein contents were significantly increased in the BALF from O_3_-exposed Tg+ versus other three groups (Figure 1B). The BALF dsDNA contents were comparable in the O_3_-exposed WT versus FA-exposed WT mice. As compared to the remaining three groups, the O_3_-exposed Tg+ mice exhibited significant increase in BALF dsDNA contents (Figure 1C).

O_3_ is known to cause lipid peroxidation (Chen et al., 2007) and alveolar macrophages are involved in the phagocytic clearance of these lipids (Dahl et al., 2007). Since the Tg+ mice possess hyper-concentrated airway surface liquid (Mall et al., 2004), we speculated that the levels of oxidized lipids will be elevated in the airspaces of O_3_-exposed Tg+ mice. Accordingly, we assessed the levels of phagocytosed lipids in alveolar macrophages from the FA- and O_3_-exposed WT and Tg+ mice. While the FA-exposed WT mice were devoid of Oil-Red-O-stained macrophages, ∼10% macrophages in the FA-exposed Tg+ mice were stained positively with Oil-Red-O staining. While O_3_-exposed WT showed ∼2% macrophages with Oil-Red-O staining, ∼21% of the alveolar macrophages from O_3_-exposed Tg+ mice were positively stained with Oil-Red-O staining (Figures 1D,E).

### 3.2 Ozone exposure disrupts the immune cell composition and alters the level of inflammatory mediators in the lung airspaces of WT and *Scnn1b*-Tg+ mice

**FIGURE 2:**
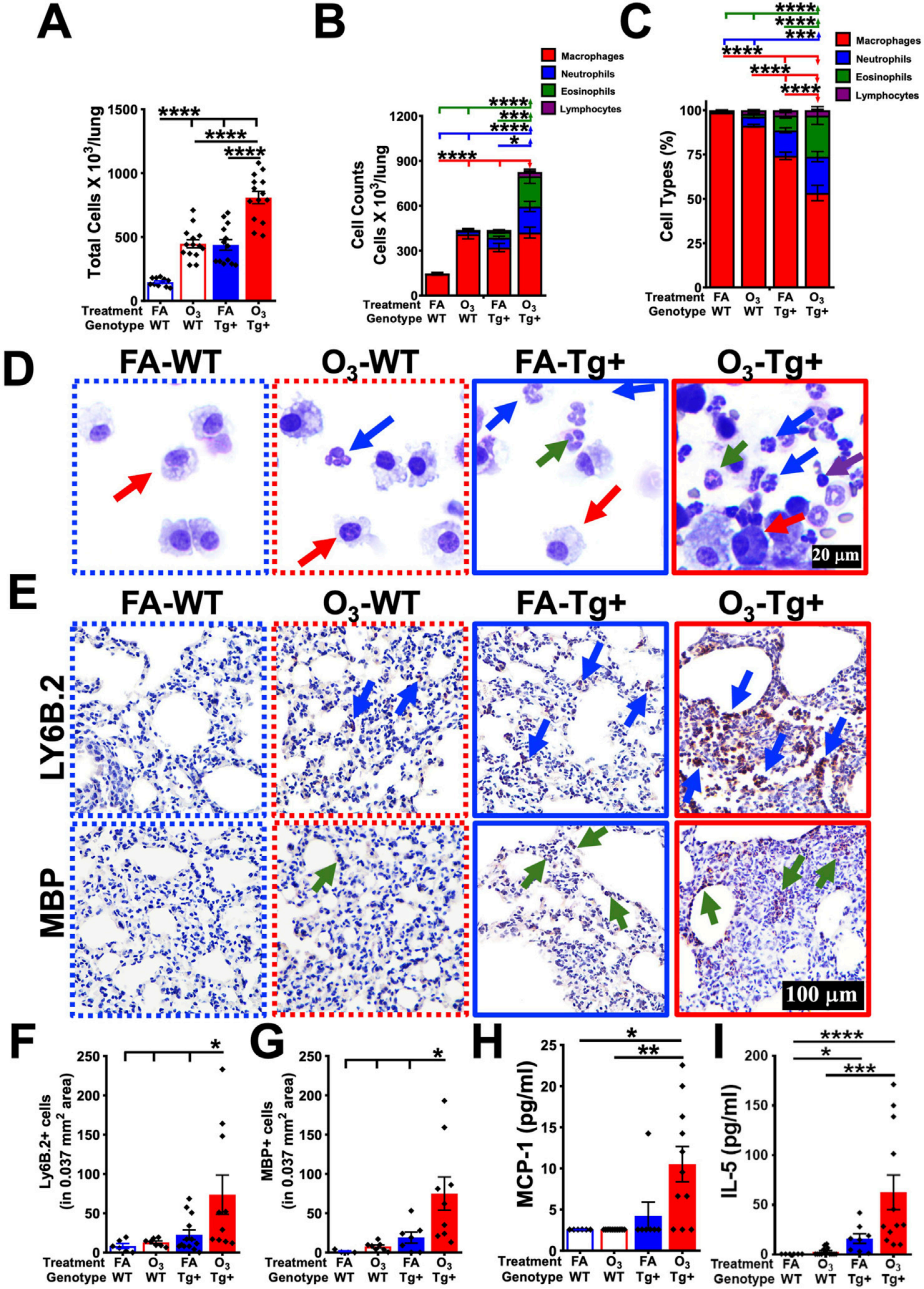
Ozone exposure disrupts the immune cell composition and alters the level of inflammatory mediators in the lung airspaces of WT and *Scnn1b*-Tg+ mice. **(A)** Total cell counts (cells x10^3^/lung) from FA-exposed WT (blue open bar), O_3_-exposed WT (red open bar), FA-exposed *Scnn1b*-Tg+ (blue solid bar), and O_3_-exposed *Scnn1b*-Tg+ (red solid bar) mice. Error bars represent standard error of the mean (SEM), ****p < 0.0001 using one-way ANOVA, followed by Tukey’s multiple comparison *post hoc* test. **(B, C)** Differential cell counts in stacked bar graphs [Macrophage (red), Neutrophils (blue), Eosinophils (green), Lymphocytes (purple)]. Error bars represent SEM, *p < 0.05, ***p < 0.001, ****p < 0.0001 using one-way ANOVA, followed by Tukey’s multiple comparison *post hoc* test. **(D)** Representative photomicrographs of Wright-Giemsa-stained BALF cytospin from FA-exposed WT (blue dotted border), O_3_-exposed WT (red dotted border), FA-exposed *Scnn1b*-Tg+ (blue solid border), and O_3_-exposed *Scnn1b*-Tg+ (red solid border) mice [Macrophages (red arrows), Neutrophils (blue arrows), Eosinophils (green arrows), Lymphocytes (purple arrows)]. All photomicrographs were taken at same magnification. **(E)** Representative photomicrographs of Lymphocyte antigen 6B.2 (Ly-6B.2) (upper panels) and Major Basic Protein (MBP) (bottom panels)-stained lung sections from FA-exposed WT (blue dotted border), O_3_-exposed WT (red dotted border), FA-exposed *Scnn1b*-Tg+ (blue solid border), and O_3_-exposed *Scnn1b*-Tg+ (red solid border) mice. Blue arrows depict neutrophils positively stained for Ly-6B.2; green arrows depict eosinophils positively stained for MBP. All photomicrographs were taken at same magnification. Fiji quantification of **(F)** Ly-6B.2- and **(G)** MBP-stained cells per unit area analyzed in tissue sections from FA-exposed WT (blue dotted border), O_3_-exposed WT (red dotted border), FA-exposed *Scnn1b*-Tg+ (blue solid border), and O_3_-exposed *Scnn1b*-Tg+ (red solid border) mice. Error bars represent SEM, *p < 0.05 using one-way ANOVA, followed by Tukey’s multiple comparison *post hoc* test. Cytokine levels (pg/ml; picogram per milliliter) of **(H)** monocyte chemoattractant protein-1 (MCP-1) and **(I)** interleukin-5 (IL-5) in BALF from FA-exposed WT (blue dotted border), O_3_-exposed WT (red dotted border), FA-exposed *Scnn1b*-Tg+ (blue solid border), and O_3_-exposed *Scnn1b*-Tg+ (red solid border) mice. Error bars represent SEM, *p < 0.05, **p < 0.01, ***p < 0.001, ****p < 0.0001 using one-way ANOVA, followed by Tukey’s multiple comparison *post hoc* test.

To examine the effect of repetitive O_3_ exposure on the immune cell profiles in the lung airspaces, we performed immune cell analyses on the BALF from FA- or O_3_-exposed WT and Tg+ mice. As compared to the FA-exposed WT mice, the O_3_-exposed WT mice exhibited a significant increase of total cell counts in the BALF, which was attributable to the significant increase in macrophage counts (Figures 2A–D). While the neutrophils and eosinophils were identified in the BALF from O_3_-exposed WT versus FA-exposed WT mice, the differences were not statistically significant (Figures 2A–D). As compared to the FA-exposed Tg+ mice, the O_3_-exposed Tg+ mice exhibited a significant increase of total cell counts in the BALF, which was attributable to the significant increase in neutrophil and eosinophil counts (Figures 2A–D).

To further assess the presence of inflammatory cells in the lung tissues, immunohistochemical analyses were performed for neutrophils, i.e., Lymphocyte antigen 6B.2 (Ly-6B.2) and eosinophils, i.e., Major Basic Protein (MBP) (Figures 2E–G). Consistent with the increased BALF granulocytic counts in O_3_-exposed Tg+ mice, the tissue staining of Ly-6B.2 and MBP was markedly increased in O_3_-exposed Tg+ mice (Figures 2E–G). These data suggest that O_3_ exposure exaggerates the recruitment of immune cells to the Tg+ mice lungs.

To determine the correlations between the immune cell infiltrations and the levels of immune cell chemoattractants in the airspaces of O_3_-exposed WT and Tg+ mice, we assayed the levels of chemoattractants in the cell-free BALF of FA- and O_3_-exposed WT and Tg+ mice. The levels of monocyte chemoattractant protein-1 (MCP-1/CCL2) and interleukin-5 (IL-5) were comparable between the BALF of O_3_-exposed versus FA-exposed WT mice (Figures 2H,I). The increase in neutrophil and eosinophil counts in the BALF and lung tissue was associated with the significantly increased levels of monocyte chemoattractant protein-1 (MCP-1/CCL2) and interleukin-5 (IL-5) in the BALF of O_3_-exposed versus FA-exposed Tg+ mice (Figures 2H,I). Additional data for other inflammatory mediators are included in Supplementary Figure S1. The BALF levels of granulocyte-colony stimulating factor (G-CSF), keratinocyte chemoattractant (KC/CXCL1), and macrophage inflammatory protein 2 (MIP-2/CXCL2), responsible for neutrophils production, recruitment, and activation, were comparable in FA- and O_3_-exposed Tg+ mice (Supplementary Figures S1A–C). While the levels of macrophage inflammatory protein 1-alpha (MIP-1α/CCL3), a chemoattractant for innate immune cells including macrophages, eosinophils, neutrophils, and lymphocytes, were decreased in O_3_-exposed Tg+ mice versus FA-exposed Tg+ mice (Supplementary Figure S1D), the levels of macrophage inflammatory protein 1-beta (MIP-1β/CCL4) were comparable between FA- and O_3_-exposed Tg+ mice (Supplementary Figure S1E). On the other hand, the levels of pro-inflammatory cytokines, i.e., interleukin-1 alpha (IL-1α), and tumor necrosis factor alpha (TNF-α), were decreased in O_3_-exposed Tg+ mice versus FA-exposed Tg+ mice (Supplementary Figures S1F, G). The levels of interleukin-6 (IL-6), C-X-C motif chemokine 10 (CXCL10/IP-10), and interleukin-17 (IL-17), consistently found in chronic and sub-chronic O_3_-exposed airspaces, trended higher in O_3_-exposed Tg+ mice as compared to FA-exposed Tg+ mice (Supplementary Figures S1H–J). Of note, the levels of anti-inflammatory cytokines, i.e., interleukin-9 (IL-9), and interleukin-10 (IL-10) were significantly decreased in O_3_-exposed Tg+ mice as compared to FA-exposed Tg+ mice (Supplementary Figures S1K, L).

### 3.3 Ozone exposure exacerbates lung pathology in WT and *Scnn1b*-Tg+ mice

**FIGURE 3:**
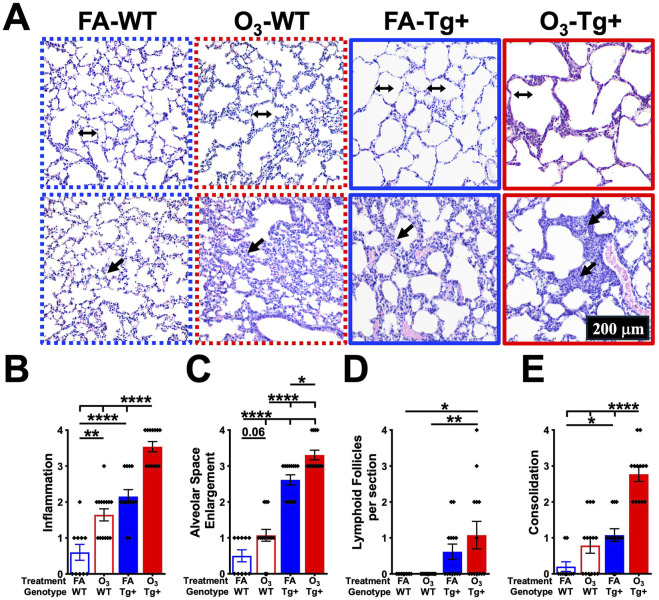
Ozone exposure exacerbates lung pathology in WT and *Scnn1b*-Tg+ mice. **(A)** Representative photomicrographs of Hematoxylin and Eosin (H&E)-stained lung sections from FA-exposed WT (blue dotted border), O_3_-exposed WT (red dotted border), FA-exposed *Scnn1b*-Tg+ (blue solid border), and O_3_-exposed *Scnn1b*-Tg+ (red solid border) mice. Double-headed arrows (all with similar dimensions) depict linear alveolar space width; black arrows depict immune cell infiltration in peribronchiolar spaces. All photomicrographs were taken at same magnification. Semiquantitative histopathological scores for **(B)** inflammation, **(C)** alveolar space enlargement, **(D)** lymphoid follicles per section, and **(E)** consolidation in FA-exposed WT (blue open bar), O_3_-exposed WT (red open bar), FA-exposed *Scnn1b*-Tg+ (blue solid bar), and O_3_-exposed *Scnn1b*-Tg+ (red solid bar) mice. Error bars represent standard error of the mean (SEM), *p < 0.05, **p < 0.01, ***p < 0.001, ****p < 0.0001 using one-way ANOVA, followed by Tukey’s multiple comparison *post hoc* test.

The alterations in lung architecture and pathology associated with the inflammatory changes of *Scnn1b*-Tg+ mice following O_3_ exposure were evaluated. As compared with the FA-exposed WT mice, the O_3_-exposed WT mice displayed perivascular and peribronchiolar inflammation, alveolar space enlargement, and consolidation around alveolar septa (Figures 3A–E). As previously reported (Lewis et al., 2017; Choudhary et al., 2021c), the FA-exposed Tg+ mice had significant perivascular and peribronchiolar inflammation, alveolar space enlargement, and consolidation (Figures 3A–E). As compared with FA-exposed Tg+ mice, the O_3_-exposed Tg+ mice exhibited significantly increased perivascular and peribronchiolar inflammation, alveolar space enlargement, and septal thickening/consolidation (Figures 3A–E). Notably, the number of lymphoid follicles per section were not different between FA- and O_3_-exposed Tg+ mice (Figures 3A–E).

### 3.4 Ozone exposure increases MMP12 production in *Scnn1b*-Tg+ mice

**FIGURE 4:**
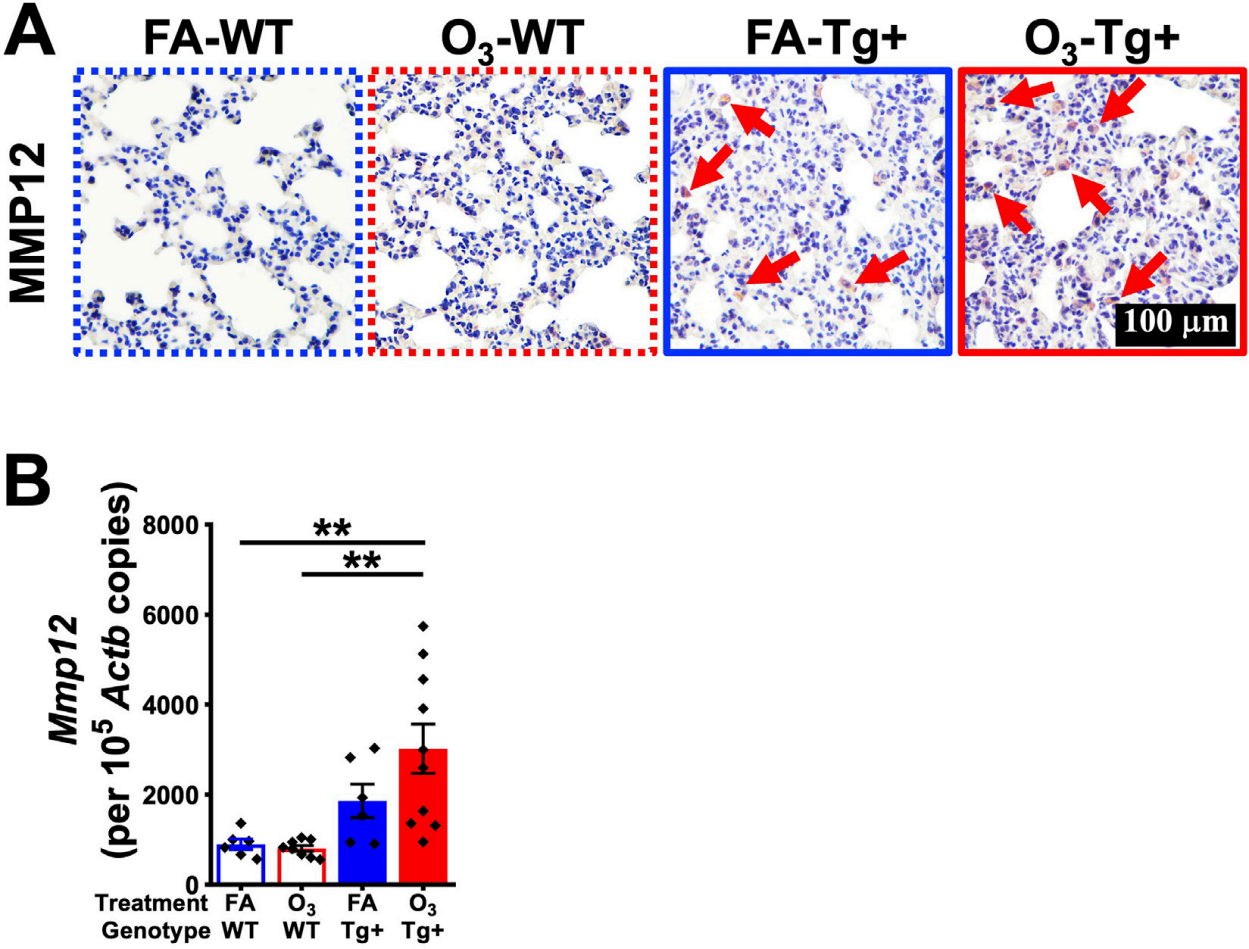
Ozone exposure increases MMP12 production in *Scnn1b*-Tg+ mice. **(A)** Representative photomicrographs of MMP12-stained lung sections from FA-exposed WT (blue dotted border), O_3_-exposed WT (red dotted border), FA-exposed *Scnn1b*-Tg+ (blue solid border), and O_3_-exposed *Scnn1b*-Tg+ (red solid border) mice. Red arrows depict cells positively stained for MMP12. All photomicrographs were taken at same magnification. **(B)** Absolute quantification of *Mmp12* mRNA in FA-exposed WT (blue open bar), O_3_-exposed WT (red open bar), FA-exposed *Scnn1b*-Tg+ (blue solid bar), and O_3_-exposed *Scnn1b*-Tg+ (red solid bar) mice. Error bars represent standard error of the mean (SEM), **p < 0.01 using one-way ANOVA, followed by Tukey’s multiple comparison *post hoc* test.

MMP12 is a macrophage metalloprotease responsible for the proteolytic attack on the alveolar wall matrix, resulting in emphysema (Finlay et al., 1997; Hendrix and Kheradmand, 2017). MMP12 is critical in the formation of emphysema in *Scnn1b*-Tg+ model (Trojanek et al., 2014). To determine whether the enhanced emphysematous responses in the O_3_-exposed Tg+ mice are caused by the upregulated expression of MMP12, we assessed MMP12 protein contents and *Mmp12* mRNA levels in the lungs of FA- and O_3_-exposed mice. MMP12 protein contents and *Mmp12* mRNA levels were comparable between the lungs of FA- and O_3_-exposed WT mice (Figures 4A,B). The O_3_-exposed Tg+ mice had significantly increased MMP12+ cell counts versus FA-exposed Tg+ mice (Figures 4A,B). Furthermore, the MMP12+ cells staining intensity were enhanced in O_3_-exposed Tg+ mice as compared to FA-exposed Tg+ mice (Figures 4A,B). These data suggest that O_3_ exposure may enhance MMP12 activity in *Scnn1b*-Tg+ mice and result in exaggerated alveolar space enlargement (Figures 4A,B). Consistent with MMP12 immunostaining, the *Mmp12* mRNA levels were increased in O_3_-exposed Tg+ mice versus FA-exposed Tg+ mice, but the differences were not statistically significant (Figure 4B).

### 3.5 Ozone does not exacerbate mucus obstruction and mucous cell metaplasia in *Scnn1b*-Tg+ mice

**FIGURE 5:**
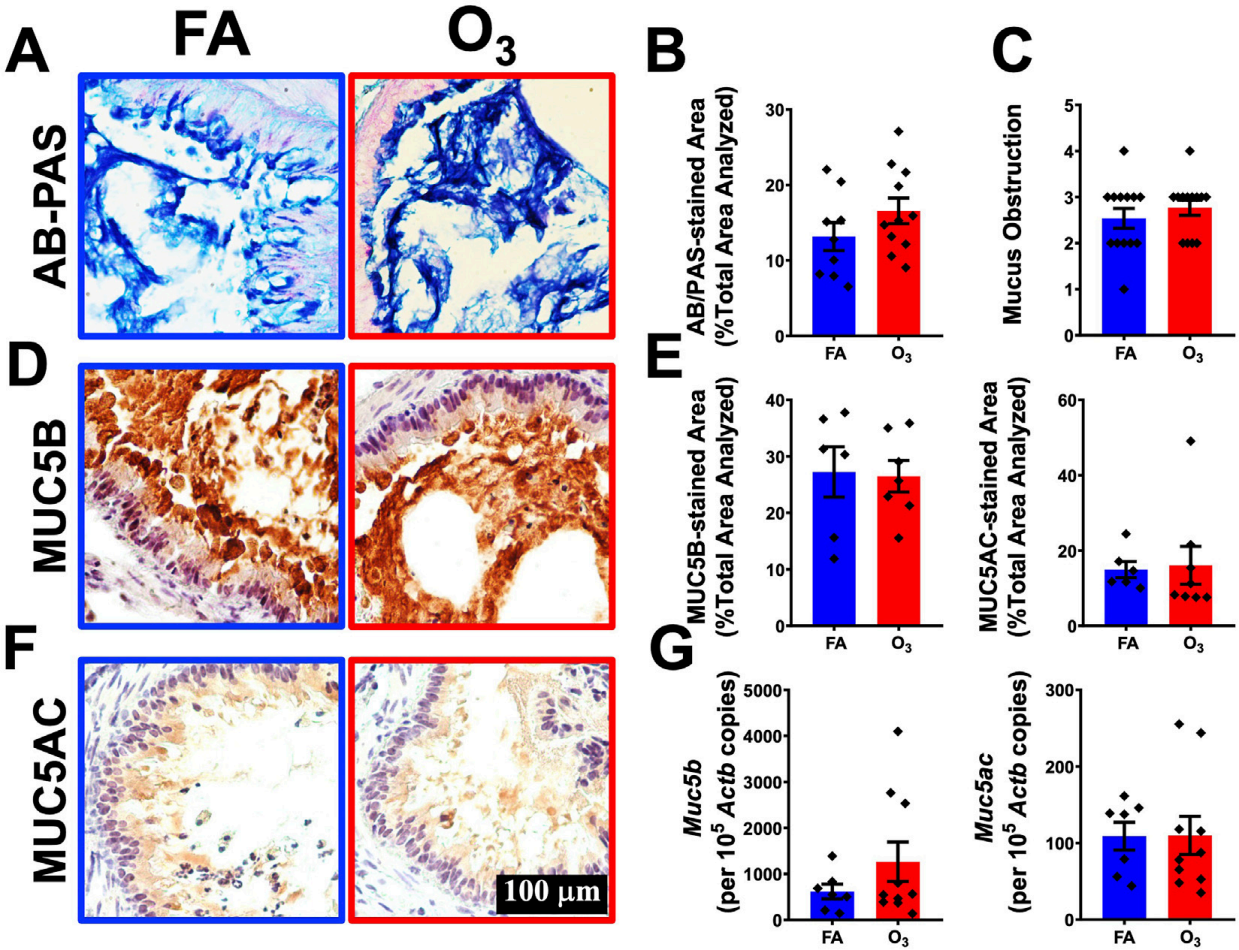
Ozone does not exacerbate mucus obstruction and mucous cell metaplasia in *Scnn1b*-Tg+ mice **(A)** Representative photomicrograph of alcian blue/periodic acid-Schiff (AB/PAS)-stained lung sections from FA-exposed *Scnn1b*-Tg+ (blue solid border), and O_3_-exposed *Scnn1b*-Tg+ (red solid border) mice. All photomicrographs were taken at same magnification. **(B)** Quantification of AB/PAS-stained cells per unit area analyzed in tissue sections from FA-exposed *Scnn1b*-Tg+ (blue solid bar), and O_3_-exposed *Scnn1b*-Tg+ (red solid bar) mice. Error bars represent standard error of the mean (SEM) using Student’s t-test. **(C)** Semiquantitative histopathological scores for mucus obstruction in FA-exposed *Scnn1b*-Tg+ (blue solid bar), and O_3_-exposed *Scnn1b*-Tg+ (red solid bar) mice. Error bars represent SEM, using Student’s t-test. **(D)** Representative photomicrograph of MUC5B-stained lung sections from FA-exposed *Scnn1b*-Tg+ (blue solid border), and O_3_-exposed *Scnn1b*-Tg+ (red solid border) mice. All photomicrographs were taken at same magnification. **(E)** Quantification of MUC5B (left panel), and MUC5AC (right panel)-stained cells per unit area analyzed in tissue sections from FA-exposed *Scnn1b*-Tg+ (blue solid bar), and O_3_-exposed *Scnn1b*-Tg+ (red solid bar) mice. Error bars represent SEM using Student’s t-test. **(F)** Representative photomicrograph of MUC5AC-stained lung sections from FA-exposed *Scnn1b*-Tg+ (blue solid border), and O_3_-exposed *Scnn1b*-Tg+ (red solid border) mice. **(G)** Absolute quantification of *Muc5b* (left panel) and *Muc5ac* (right panel) mRNA in FA-exposed *Scnn1b*-Tg+ (blue solid bar), and O_3_-exposed *Scnn1b*-Tg+ (red solid bar) mice. Error bars represent SEM using Student’s t-test.

Airway mucus obstruction is a hallmark feature of human CF and CF-like lung disease in *Scnn1b*-Tg+ mouse model (Choudhary et al., 2021c; Lewis et al., 2020a; 2020b; 2017; Mall et al., 2004; Mao et al., 2022). To examine the effects of O_3_ on the progression of mucus obstruction, we used Alcian Blue-PAS (AB-PAS) staining to access the mucous cell metaplasia (MCM) and mucus obstruction status in the airways of FA- and O_3_-exposed *Scnn1b*-Tg+ mice. The MCM and mucus obstruction levels were comparable in O_3_-exposed as compared to FA-exposed Tg+ mice (Figures 5A–C).

MUC5B and MUC5AC are the most common gel forming mucins in the airways of human CF and CF-like lung disease mouse model (Livraghi-Butrico et al., 2017). Therefore, to examine the role of O_3_ in the expression of these mucin-forming proteins, we performed immunolocalization of MUC5B and MUC5AC in the lung sections of FA- and O_3_-exposed Tg+ mice. The immunostaining MUC5B and MUC5AC was comparable between in O_3_-exposed and FA-exposed Tg+ mice (Figures 5D,E). Consistent with the immunohistochemistry data, gene expression data of mucin genes, i. e., *Muc5ac* and *Muc5b* showed no difference between FA- and O_3_-exposed Tg+ mice (Figures 5F,G). Of note O_3_ exposure induces a significant upregulation of Resistin-like alpha (RETNLA), a M2 macrophage protein marker, in the airway epithelial cells and macrophages of adult mice (Choudhary et al., 2021b). Consistently, in the current study, we observed RETNLA-expressing cells were significantly elevated in the lung sections of O_3_-exposed versus FA-exposed Tg+ mice (Supplementary Figure S2).

## 4 Discussion

The initiation of ozone (O_3_)-induced airway injury involves the ozonation of proteins, lipids, and other metabolite contents in the airway surface liquid (ASL) (Patial and Saini, 2020). In human cystic fibrosis (CF) lung disease, the ASL dehydration causes the hyperconcentration of various solids that are present in the airways, which results in various outcomes including compromised mucociliary function, mucin hypersecretion, mucus obstruction, and airway inflammation (Lewis et al., 2019). The *Scnn1b*-Tg+ (Tg+) mice, due to ion channel dysfunction-mediated ASL dehydration, manifest most of the CF lung disease features (Mall et al., 2004). Our previous reports demonstrated remarkable similarities between the lung disturbances caused due to the ASL dehydration in Tg+ mice and sub-chronic O_3_-induced lung disease in C57BL/6J mice (Choudhary et al., 2021a; 2021b). These similarities led us to test our hypothesis that the superimposition of O_3_ exposure on Tg+ muco-inflammatory disease will result in the worsening of Tg+ lung disease in the developing postnatal lung (Choudhary et al., 2021c). Our investigation revealed interesting findings on how the O_3_ exposure impacts the pathological features of Tg+ lung disease (Choudhary et al., 2021c). However, the effects of O_3_ exposure in the developed lungs of *Scnn1b*-Tg+ mice remained untested.

In this study, we focused our investigation into the interactions between O_3_ exposure and muco-inflammatory airway disease during the postweaning period, when the lung development is almost complete (Bartman et al., 2020; Rackley and Stripp, 2012). O_3_ is a highly reactive but less water-soluble gas (Gerrity et al., 1995). Before reaching lung epithelium, O_3_ molecules interact with the constituents in the ASL, such as proteins, lipids, antioxidants, and immune cells (Patial and Saini, 2020). Therefore, the cellular damage caused by O_3_ is less likely a direct result of its interaction with epithelial cells, and is more likely mediated by the action of various ozonation products (Patial and Saini, 2020). *Scnn1b*-Tg+ mice exhibit increased concentration of percent solids in the ASL layer, which are likely the immediate targets of ozonation. Therefore, we speculated that, as compared with the WT mice, the *Scnn1b*-Tg+ mice would be exposed to higher levels of ozonated ASL contents and, consequently, display amplified inflammatory responses.

In the current study, we observed a significant increase in total protein and dsDNA contents in O_3_-exposed Tg+ mice as compared to FA-exposed Tg+ mice. Repetitive O_3_ exposure has been reported to be associated with epithelial cell death (Triantaphyllopoulos et al., 2011). It is likely that O_3_ causes extensive cellular necrosis to the airway epithelium of *Scnn1b*-Tg+ mice, resulting in the marked increase of dsDNA and protein contents. The observed elevated total protein contents in O_3_-exposed *Scnn1b*-Tg+ mice can also be attributed to other factors, e.g., disruption of the integrity of the gas exchange barrier, resulting in the leakage of proteins into the alveolar airspaces (Currie et al., 1998; Michaudel et al., 2018), excessive release of protein products from recruited cells, i.e., eosinophil granule proteins (Jacobsen et al., 2017) and the formation of neutrophil extracellular traps (NETs) (Xu et al., 2024).

Alveolar space enlargement is a consistent feature in *Scnn1b*-Tg+ mouse model (Choudhary et al., 2021c; Mall et al., 2008), which is associated with the release of MMP12, a matrix metalloproteinase, by activated airway macrophages (Trojanek et al., 2014). MMP12 causes alveolar wall destruction, leading to alveolar space enlargement (Finlay et al., 1997; Hendrix and Kheradmand, 2017). MMP12 is expressed in alveolar macrophages of smokers with chronic obstructive pulmonary disease (COPD), but is rarely detected in healthy macrophages (Babusyte et al., 2007). In the current study, we observed MMP12-expressing cells in the lung tissue from FA-exposed Tg+ mice. MMP12 has been reported to be an important mediator in the pathogenesis of both acute and chronic lung injury (Hautamaki et al., 1997; Nénan et al., 2007; Warner et al., 2001). Disruption of *Mmp12* gene resulted in reduced total BAL protein, reduced neutrophil infiltration into the lung airspaces and reduced lung injury following an immune complex-induced acute lung injury (Warner et al., 2001). Moreover, studies using MMP12 knockout mice have demonstrated that MMP12 is linked to the development of alveolar space enlargement following chronic cigarette smoke exposure (Hautamaki et al., 1997). Additionally, a single instillation of recombinant human MMP12 (rhMMP12) was used to test the direct effect of MMP12 in the development of the inflammatory process in mouse airways. Accordingly, rhMMP12 induced an acute recruitment of neutrophils that was associated with the increased of pro-inflammatory mediators (Nénan et al., 2007). In the current study, the O_3_ exposure of *Scnn1b*-Tg+ lung disease resulted in alveolar space enlargement, which is likely an outcome of enhanced macrophage and neutrophil recruitment, and an increase in MMP12-expressing macrophages.

Longitudinal BAL *Scnn1b*-Tg+ studies demonstrated that airway inflammation is characterized by the early influx of neutrophils (∼PND5), succeeded by the influx of macrophages and eosinophils (∼PND14). These leucocyte influxes are waned when *Scnn1b*-Tg+ mice reach juvenile stage (Zhou et al., 2011). This is consistent with our findings, where FA-exposed Tg+ mice did not exhibit extensive neutrophils and eosinophils recruitment that were observed in their neonatal counterparts (Choudhary et al., 2021c). These differences in granulocytic infiltration suggest that developed Tg+ lungs are less sensitive to O_3_-induced granulocytic recuitment as compared to underdeveloped neonatal Tg+ lungs. Additionally, O_3_ exposure worsened the muco-inflammatory features of neonatal *Scnn1b*-Tg+, i.e., granulocytic (neutrophilic and eosinophilic) recruitment, perivascular and peribronchiolar inflammation, alveolar space enlargement, septal thickening/consolidation, and mucus obstruction (Choudhary et al., 2021c). However, in this current study, while granulocytic recruitment, perivascular and peribronchiolar inflammation, alveolar space enlargement, septal thickening/consolidation were present in O_3_-exposed Tg+ mice, mucus obstruction and production of major gel-forming mucins, i.e., MUC5B and MUC5AC were not different between FA-exposed and O_3_-exposed Tg+ mice.

It was previously demonstrated that the lungs of Tg+ mice and O_3_-exposed mice share key immunopathological features (Mall et al., 2004; Choudhary et al., 2021a; Choudhary et al., 2021b; Choudhary et al., 2021c). For instance: 1) airway inflammation, as evidenced by the increased granulocytic recruitment; 2) increased number of macrophages; 3) increased levels of inflammatory mediators; and 4) enhanced muco-inflammatory responses. Alveolar macrophages (AMs), sentinel cells in the airspace at homeostasis, patrol the epithelial surfaces of the pulmonary airway and alveolar spaces and face the continued onslaught from inhaled environmental insults (Hussell and Bell, 2014). Thus, we speculated that AMs play a critical role in regulating O_3_-induced lung injury. An earlier study in mice exposed to O_3_ showed that macrophages expressed increased levels of MCP-1, a potent neutrophil and macrophage chemoattractant (Zhao et al., 1998), consistent with the observed increased in the levels of MCP-1 in the BALF of O_3_-exposed Tg+ mice as compared to FA-exposed Tg+ mice. Additionally, mucus obstruction is a consistent feature of *Scnn1b*-Tg+ lung disease (Choudhary et al., 2021c; Lewis et al., 2020a; 2020b; 2017; Mall et al., 2004; Mao et al., 2022). Gel-forming mucins, i.e., MUC5B and MUC5AC were previously found to be enriched in the exosomes of O_3_-exposed C57BL/6J mice (Choudhary et al., 2021b). Consistent with these reports, a study using neonatal *Scnn1b*-Tg+ mouse model demonstrated the marked increase in mucus obstruction levels, accompanied by the increased levels of MUC5B and MUC5AC in O_3_-exposed Tg+ mice (Choudhary et al., 2021c). However, in current study, while AB-PAS data showed that the levels of mucus obstruction trended higher in O_3_-exposed Tg+ mice, the levels of MUC5B and MUC5AC were not significantly different between O_3_-exposed and FA-exposed Tg+ mice. While these findings suggest similarities in muco-inflammatory responses between Tg+ and O_3_ exposed mice, the severity of the injury and the effect of lung development stage reveal distinct features.

Most of the assayed cytokines, e.g., C-X-C motif chemokine 10 (CXCL10/IP-10), macrophage inflammatory protein 1-beta (MIP-1β/CCL4), interleukin-6 (IL-6), keratinocyte chemoattractant (KC/CXCL1), granulocyte-colony stimulating factor (G-CSF), macrophage inflammatory protein 2 (MIP-2/CXCL2), and interleukin-5 (IL-5) levels, showed consistent trend with our previous report (Choudhary et al., 2021c). Interestingly, interleukin-10 (IL-10) showed opposite trend, i.e., the BALF levels of IL-10 were significantly lower in O_3_-exposed Tg+ mice. IL-10 is an anti-inflammatory mediator, mainly produced by T regulatory cells (Tregs) (Fiorentino et al., 1991), and IL-10 deficiency results in exaggerated inflammation following O_3_-induced lung injury (Backus et al., 2010). Whether the reduced levels of IL-10 cause exaggerated inflammation in O_3_-exposed Tg+ mice require further investigation.

**FIGURE 6:**
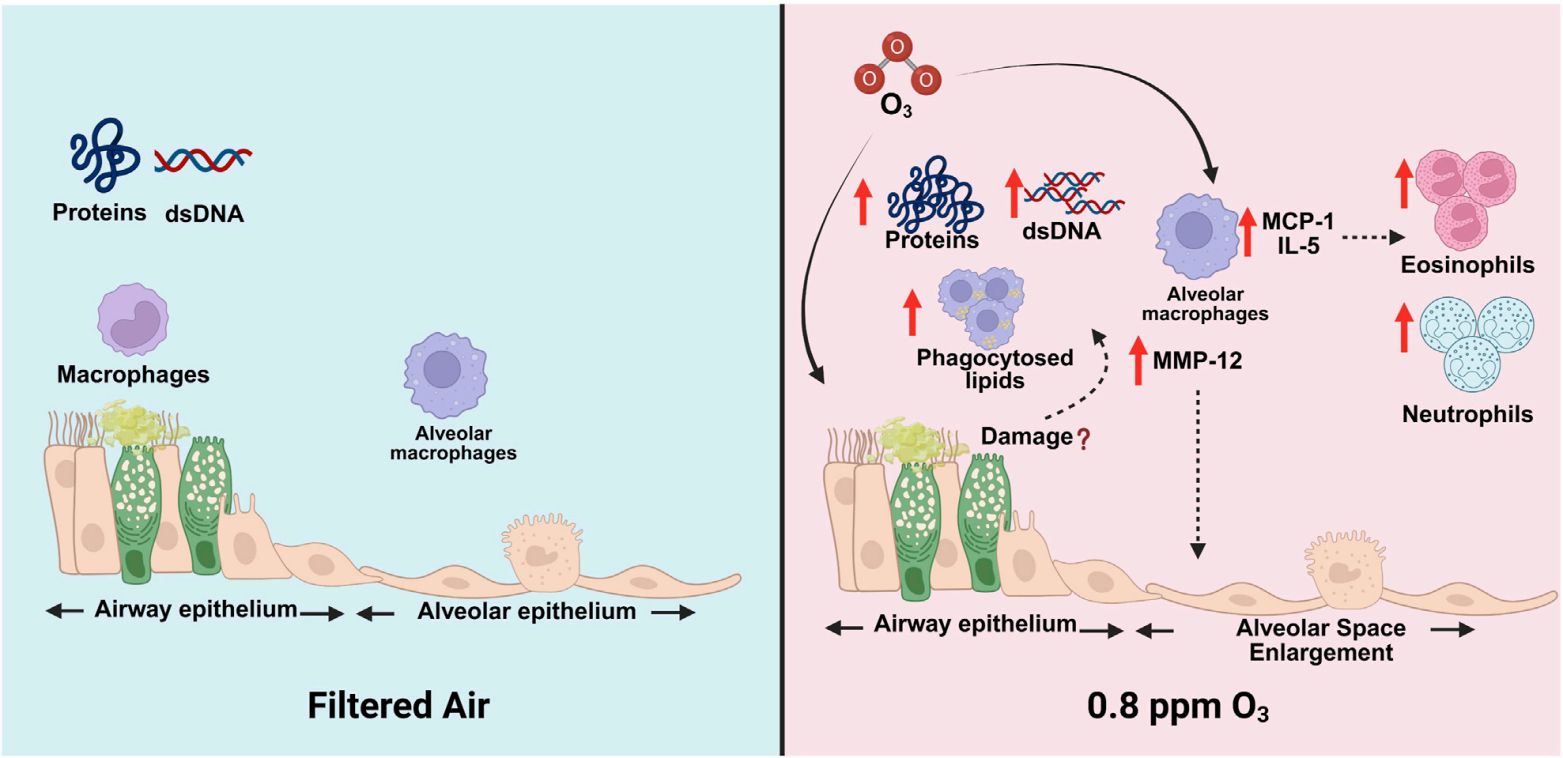
Diagrammatic summary of the ozone-induced alterations in muco-inflammatory responses in Tg+ mice.

Our data suggest that exposure to O_3_ results in increased total protein contents, total dsDNA contents, and phagocytosed lipids in Tg+ mice (Figure 6). Additionally, O_3_ enhances immune cell recruitment in Tg+ mice (Figure 6). Furthermore, O_3_ exacerbates inflammation, enhances alveolar space enlargement and tissue consolidation, but not lymphoid follicles formation in the lungs of Tg+ mice (Figure 6). Interestingly, however, as opposed to the previous report in O_3_-exposed neonatal Tg+ mice (Choudhary et al., 2021c), where the hallmark features of Tg+ airway disease, i.e., mucus obstruction and expression of major gel-forming mucins (MUC5B and MUC5AC) were found exacerbated by O_3_ exposure, the FA- and O_3_-exposed Tg+ in this study exhibited comparable responses. Collectively, this study demonstrates that O_3_ exacerbates key features of muco-inflammatory lung diseases, with responses to O_3_ exposure potentially resulting in different outcomes between young individuals, whose lungs are still developing, and adults with matured lungs.

## Data Availability

The original contributions presented in the study are included in the article/Supplementary Material, further inquiries can be directed to the corresponding author.

## Supplementary material

SUPPLEMENTARY FIGURE S1 Ozone alters the level of inflammatory mediators in the airspaces of WT and Scnn*1b-*Tg+ mice. Cytokine levels (pg/ml; picogram per milliliter) of **(A)** granulocyte-colony stimulating factor (G-CSF), **(B)** macrophage inflammatory protein 2 (MIP-2/CXCL2), **(C)** keratinocyte chemoattractant (KC/ CXCL1), **(D)** macrophage inflammatory protein 1-alpha (MIP-1α/CCL3), **(E)** macrophage inflammatory protein 1-beta (MIP-1β/CCL4), **(F)** interleukin-1 alpha (IL-1α), **(G)** interleukin-6 (IL-6), **(H)** C-X-C motif chemokine 10 (CXCL10/IP-10), **(I)** tumor necrosis factor alpha (TNF-α), **(J)** interleukin-17 (IL-17), **(K)** interleukin-9 (IL-9), **(L)** interleukin-10 (IL-10) in the BALF from FA-exposed WT (blue open bar), O_3_-exposed WT (red open bar), FA-exposed *Scnn1b*-Tg+ (blue solid bar), and O_3_-exposed *Scnn1b*-Tg+ (red solid bar) mice. Error bars represent standard error of the mean (SEM), **p* < 0.05, ***p* < 0.01, ****p* < 0.001, *****p* < 0.0001 using one-way ANOVA, followed by Tukey’s multiple comparison post hoc test.

SUPPLEMENTARY FIGURE S2 Ozone increases RETNLA expression in the airspaces of WT and *Scnn1b-*Tg+ mice. **(A)** Representative photomicrographs of RETNLA-stained lung sections from FA-exposed WT (blue dotted border), O_3_-exposed WT (red dotted border), FA-exposed *Scnn1b*-Tg+ (blue solid border), and O_3_-exposed *Scnn1b*-Tg+ (red solid border) mice. Red arrows depict alveolar macrophages positively stained for RETNLA. All photomicrographs were taken at same magnification. **(B)** Quantification of RETNLA-stained cells per unit area analyzed in tissue sections from FA-exposed WT (blue open bar), O_3_-exposed WT (red open bar), FA-exposed *Scnn1b*-Tg+ (blue solid bar), and O_3_-exposed *Scnn1b*-Tg+ (red solid bar) mice. Error bars represent standard error of the mean (SEM), ****p* < 0.001, *****p* < 0.0001 using one-way ANOVA, followed by Tukey’s multiple comparison post hoc test.
