# Supplementary figures and images for: Repetitive ozone exposure worsens features of muco-inflammatory disease in developed Scnn1b-Tg+ mice lungs
Source: Front Toxicol. 2025 May 27;7:1540468. doi: 10.3389/ftox.2025.1540468 (PMC12148915; doi:10.3389/ftox.2025.1540468)

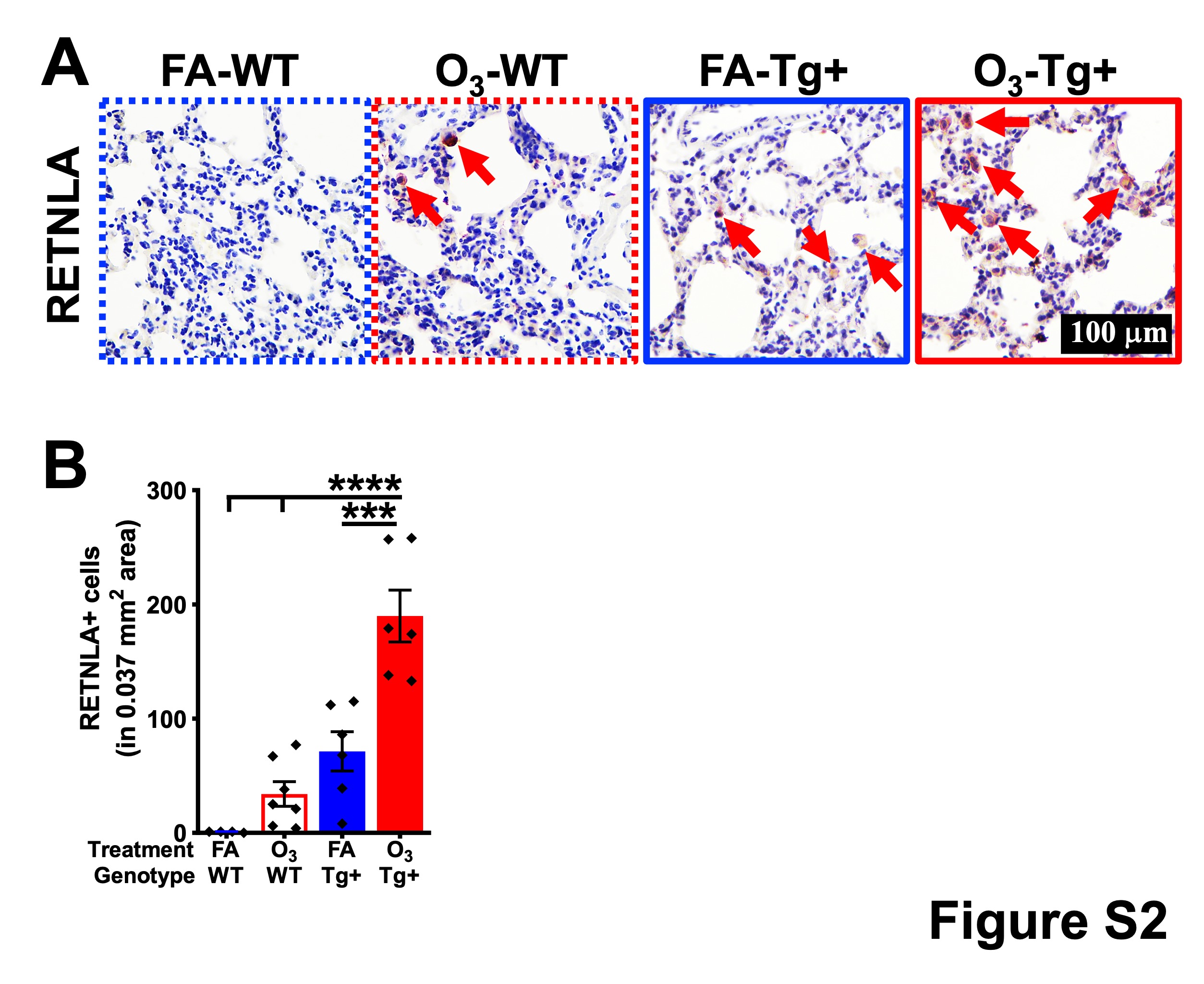

Supplement: Supplementary file 1 [file Image2.jpeg]

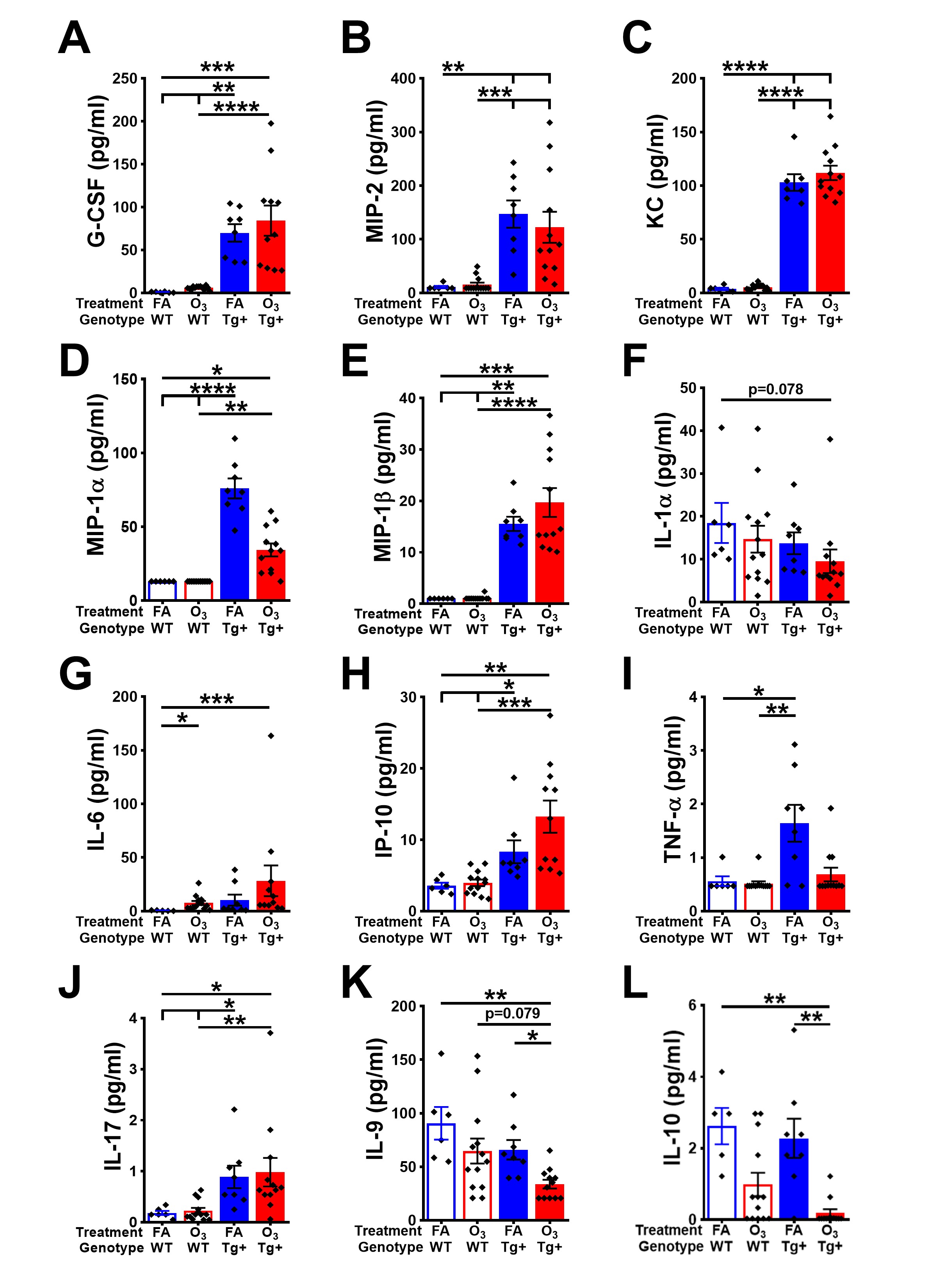

Supplement: Supplementary file 2 [file Image1.jpg]
